# Development and Validation of a Liquid Chromatography-Mass Spectrometry Method for the Determination of Zileuton in Human Plasma

**DOI:** 10.3797/scipharm.1402-19

**Published:** 2014-03-26

**Authors:** Katakam Prakash, Shanta K Adiki, Rama Rao Kalakuntla

**Affiliations:** ^1^ Nirmala College of Pharmacy, Mangalagiri, Guntur 522503, AP, India.; ^2^ Department of Biotechnology, Acharya Nagarjuna University, Guntur, AP, India.

**Keywords:** Zileuton, Plasma, LC-MS/MS, Validation

## Abstract

A selective and sensitive liquid chromatography-tandem mass spectrometric method (LC-MS/MS) has been developed and validated for the quantification of zileuton in human plasma. Deuterated internal standard (zileuton D4) was used as the internal standard (ISTD). Zileuton was extracted by liquid-liquid extraction using methyl *tert*-butyl ether and separated by isocratic elution on a C18 column (100 × 4.6 mm, 5 μm, Discovery C18) with the mobile phase consisting of 1 mM ammonium acetate buffer and methanol in the ratio of 10:90. A flow rate of 1.0 ml/min was used with isocratic elution. Multiple reaction monitoring transitions in positive mode for zileuton and the internal standard were 237.3/161.2 and 241.2/161.1, respectively. The method was validated within the linearity range of 50.5–10,012.7 ng/ml for the bioanalytical method validation parameters like selectivity, accuracy, precision, recovery, stability, and matrix effect.

## Introduction

**Fig. 1.**
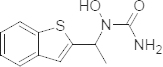
Chemical structure of zileuton

Zileuton is an asthma drug that differs chemically and pharmacologically from other antiasthmatic agents. Zileuton is chemically known as 1-[1-(1-benzothiophen-2-yl)ethyl]-1-hydroxyurea (Figure 1) and is an orally active inhibitor of 5-lipoxygenase, the enzyme that catalyzes the formation of leukotrienes from arachidonic acid. Leukotrienes are substances that induce numerous biological effects including the augmentation of neutrophil and eosinophil migration, neutrophil and monocyte aggregation, leukocyte adhesion, increased capillary permeability, and smooth muscle contraction [1, 2]. These effects contribute to inflammation, edema, mucus secretion, and bronchoconstriction in the airways of asthmatic patients. Zileuton relieves such symptoms through its selective inhibition of 5-lipoxygenase, the enzyme that catalyzes the formation of leukotrienes from arachidonic acid. Specifically, it inhibits leukotriene LTB4, LTC4, LTD4, and LTE4 formation. Due to the role of leukotrienes in the pathogenesis of asthma, modulation of leukotriene formation by interruption of 5-lipoxygenase activity may reduce airway symptoms, decrease bronchial smooth muscle tone, and improve asthma control. Zileuton is indicated for the prophylaxis and chronic treatment of asthma in adults and children 12 years of age and older [3]. The recommended dose of zileuton is one 600 mg tablet, four times per day. Extended release formulations are also available with the dosage of 1200 mg taken twice a day [4, 5]. Following oral administration, zileuton is rapidly absorbed with a mean time to peak blood serum concentration of 1.7 h. Elimination of zileuton is predominantly via metabolism with a mean terminal half-life of 2.5 h. The urinary excretion of the inactive N-dehydroxylated metabolite and unchanged zileuton each accounted for less than 0.5% of the dose. Maximal blood concentrations of patients taking the standard zileuton dosage regimen were reported as 4.4±1.0 μg/ml [6]. Zileuton’s property of inhibiting 5-lipoxygenase has been investigated for some other therapies, like for inflammatory pain [7], acne [8], sickle disease [9], ischemia [10], and cardioprotective effects [11].

Although the use of zileuton has been investigated for new therapies in addition to the approved asthma therapy, very few quantitative bioanalytical methods are reported for the determination of zileuton from the biological matrix. To the authors’ knowledge, three bioanalytical methods were reported for the determination of zileuton by HPLC [2, 12, 13] and one bioanalytical method was recently reported by using LC-MS/MS [14]. Zileuton was used as the internal standard in some of the bioanalytical methods for the determination of aminoflavone [15] and ABT-888 [16]. The reported HPLC methods are time-consuming. The LC-MS/MS method reported used the automated 96-well plates for the sample processing and gradient mobile phase for the analysis with the total run time of 4 min. The reported LC-MS/MS method established a very wide linearity range of 3 to 20,000 ng/ml using a complex quadratic curve fit, whereas the literature’s reported maximum concentration was around 4,400 ng/ml [6]. As the automated 96-well plates were used, the applicability of the method was discouraged for the pharmacokinetic/bioequivalence studies with the smaller analytical batch size.

Bioequivalence and/or pharmacokinetic studies become an integral part of generic drug applications and a simple, sensitive, reproducible validated bioanalytical method should be required for the quantification of the intended analyte. Bioequivalence studies for zileuton need to be performed with the higher dosage of 600 mg tablets or 1200 mg extended-release tablets to support the generic abbreviated new drug applications. As per regulatory guidelines for the bioanalytical method validation, simple regression should be used wherever possible and the lower limit of quantification should be less than 1/20^th^ of the expected maximum concentration [17, 18].

**Fig. 2.**
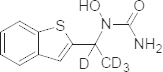
Chemical structure of zileuton D4

As the reported literature indicates the need for a simpler, faster, and more cost-effective LC-MS/MS method suitable for the quantification of zileuton for bioequivalence and/or pharmacokinetic studies, we hereby report the simple LC-MS/MS method for the determination of zileuton in human plasma using labeled internal standard (zileuton D4, Figure 2) with the short run time of 2 minutes. Sample processing employed the simple liquid-liquid extraction technique with a smaller volume of plasma (200 μl). The method was developed within the linearity range of around 50 ng/ml to 10,000 ng/ml in view of supporting the bioequivalence and/or pharmacokinetic studies of zileuton.

## Materials and Methods

### Chemicals

The zileuton reference standard was procured as a gift sample from a Pharma company and methanol (HPLC grade), ammonium acetate (GR grade), methyl *tert*-butyl ether (HPLC grade), and ammonia solution (30% pure, GR grade) were procured from Rankem, India.

### Preparation of Solutions

The ammonium acetate buffer was prepared by dissolving approximately 38 mg of ammonium acetate in 500 ml of milli-Q water and the pH of the solution was adjusted to 7.5 using the ammonia solution. A 50% methanol solution was prepared by mixing 500 ml of methanol and 500 ml of milli-Q water. The mobile phase was prepared by mixing the ammonium acetate buffer and methanol in the ratio of 10:90. Autosampler washing solution was prepared by mixing 600 ml of methanol and 400 ml of milli-Q water.

### Preparation of Standards

Zileuton and zileuton D4 stock solutions were prepared at a concentration of 2 mg/ml by dissolving in methanol and the stock solutions were stored in the refrigerator. Separate stock solutions were used for the preparation of the calibration curve and quality control samples. Spiking solutions of zileuton for the preparation of the calibration standards and quality control samples were prepared in a 50% methanol solution and spiked into the plasma at the ratio of 1:50. The calibration curve from 50.5 to 10,012.7 ng/ml was generated using eight calibration standards at the concentrations of 50.5 ng/ml (STD 1), 100.9 ng/ml (STD 2), 360.5 ng/ml (STD 3), 801.0 ng/ml (STD 4), 2,002.5 ng/ml (STD 5), 4,005.1 ng/ml (STD 6), 8,010.2 ng/ml (STD 7), and 10,012.7 ng/ml (STD 8). The quality control samples were prepared at the concentrations of 50.6 ng/ml (LLOQQC: Lower Limit of Quantification Quality Control), 148.8 ng/ml (LQC: Lower Quality Control), 4,510.4 ng/ml (MQC: Middle Quality Control), and 7,517.4 ng/ml (HQC: High Quality Control). The bulk spiked calibration standards and quality control samples were stored in the freezer. Internal standard dilution was prepared at a concentration of 800 ng/ml using the mobile phase.

### Sample Preparation and Extraction

Zileuton from the plasma was extracted using the liquid-liquid extraction technique. Flow plasma aliquot of 0.2 ml was added to the polypropylene tube containing 50 μl of the internal standard dilution and the tubes were then vortexed. Three ml of methyl *tert*-butyl ether was added and vortexed for about 10 to 15 min. After vortexing, the tubes were centrifuged at 3000 rpm for about 5 to 10 min. After centrifugation, the supernatant was transferred into the polypropylene tubes and evaporated to dryness under the stream of nitrogen at 50°C. After evaporation, the tubes were reconstituted with 0.3 ml of the mobile phase and transferred to the autosampler vials for injection.

### Chromatographic Conditions

The HPLC coupled with a mass spectrometer (LC-MS/MS; API 3000 from ABSCIEX, Canada) with the C18 column (100 × 4.6 mm, 5 μm, Discovery C18) was used and the m/z of 237.3/161.2 and 241.2/161.1 were used in multiple reaction monitoring (MRM) mode with turbo ion spray in positive mode for the quantification of zileuton and internal standard, respectively. The other mass spectrometric conditions were optimized for a reproducible response. The mobile phase used was a mixture of ammonium acetate buffer and methanol in the ratio of 10:90.

### Validation

The method performance was evaluated for selectivity, accuracy, precision, linearity, and robustness, stability during various stress conditions including benchtop stability, freezethaw stability, autosampler stability, stability of stock solutions and stock dilutions, dilution integrity, and recovery.

#### Selectivity

Selectivity was evaluated by extracting different blank plasma samples. The absence of interfering peaks at the retention time of the analyte or internal standard was considered as evidence for selectivity.

#### Linearity

Calibration curves were constructed after evaluating the linear regression for the best fit using weighing of none, 1/x, and 1/x2 for the calibration curve range of 50.5 to 10,012.7 ng/ml.

#### Recovery

Recovery of the analyte was evaluated by comparing the zileuton and internal standard response in the extracted samples versus the equivalent aqueous samples. Recovery was evaluated at three levels of quality control samples (LQC, MQC, and HQC levels). The mean recovery of the analyte and internal standard was evaluated.

#### Precision and Accuracy

For the precision and accuracy studies, samples were prepared at four concentration levels, limit of quantification (LOQQC), low (LQC), medium (MQC), and high (HQC) quality controls corresponding to 50.6, 148.8, 4,510.4, and 7517.4 ng/ml, respectively, with six replicates each. Precision and accuracy were evaluated at inter- and intraday.

#### Dilution Integrity

Dilution integrity was evaluated by diluting the sample having the concentration of approx. 15,000 ng/ml (approx. two times of the HQC) with 1/2 and 1/4 dilutions and quantified against the calibration curve to evaluate the ability to dilute the pharmacokinetic samples.

### Stability Studies

The stability of the zileuton in solutions and plasma samples was also evaluated during method validation. Zileuton stability was evaluated using two concentration levels (low and high quality control, corresponding to 50.6 and 7,517.4 ng/ml, respectively). The stability of zileuton was also evaluated in post-extracted samples kept in the autosampler at 4°C as well as in plasma samples kept in the freezer and after being stressed by freeze-thawing cycles (24 h each cycle). All samples described above were quantified using a fresh calibration curve and compared to freshly prepared quality control samples at the same concentration level.

## Results and Discussion

### Chromatographic Optimization

**Fig. 3.**
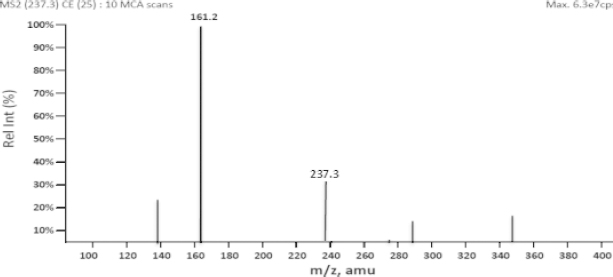
Mass spectra of zileuton

**Fig. 4.**
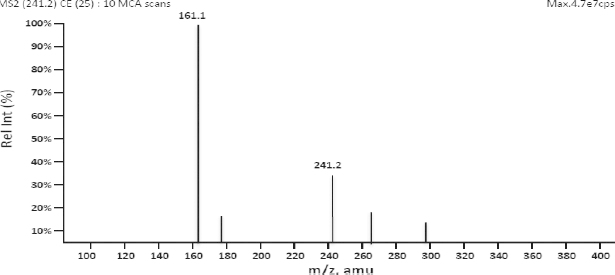
Mass spectra of zileuton D4

Liquid chromatography coupled with the mass spectrometer (LC-MS/MS) has now become a universally acceptable technique for the estimation of drugs from biological fluids as part of bioequivalence evaluations. Zileuton and the internal standard were scanned in the positive mode for the parent ion and reproducible daughter ion and the m/z ratio of 237.3/161.2 and 241.2/161.1, respectively, were selected for zileuton and the internal standard. The mass spectra of zileuton and the internal standard are presented in figures 3 and 4. The quantification was performed in multiple reaction monitoring (MRM) mode in analyst software. The compound specific mass spectrometric parameters were optimized to produce the reproducible responses for the analyte and internal standard. The mobile phase consisting of 1 mM ammonium acetate buffer and methanol in the ratio of 10:90 was optimized for better peak resolution. The ammonium acetate buffer pH was adjusted to 7.5 considering the pKa of zileuton. Chromatographic conditions were optimized to achieve good resolution and symmetric peak shape for the analyte at the lower level of quantification. The chromatographic conditions like flow rate (1.0 ml/min) and column (C18 column) conditions were also optimized with the run time of 2 min. The analyte and internal standard were quantified at 1.4 minutes. Other conditions were optimized for the reproducible quantification method.

### Sample Preparation

Zileuton was having a high partition coefficient, thus encouraging the applicability of extraction using organic solvents like protein precipitation or liquid-liquid extraction techniques. Even though protein precipitation or liquid-liquid extraction techniques are prone to cause matrix effects during mass spectrometer detection due to the usage of labeled internal standard (zileuton D4), the matrix effects shall be compensated. Protein precipitation with methanol or acetonitrile yielded very low recoveries and hence, liquid-liquid extraction was used. During liquid-liquid extraction, methyl *tert*-butyl ether (MTBE) provided optimal response and chromatography. A plasma volume of 0.2 ml was used to achieve the lower limit of quantification of 50 ng/ml.

The quality control samples were prepared at the concentrations specified in the bioanalytical method validation guidelines. The LOQQC was prepared at approximately the same concentration of the lowest calibration standard. The LQC was prepared at the concentration of less than three times of the lowest calibration standard. The MQC concentration was prepared at approximately 45% of the highest calibration standard. The HQC concentration was prepared at the concentration of approximately 75% of the highest calibration standard.

### Selectivity

**Fig. 5.**
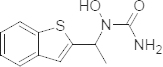
Representative chromatogram of blank plasma sample (A) zileuton and (B) zileuton D4

**Fig. 6.**
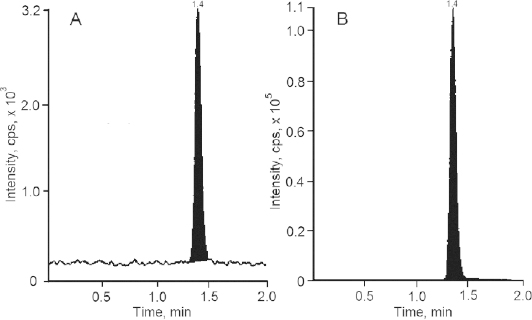
Representative chromatogram of lowest calibration standard (A) zileuton and (B) zileuton D4

**Fig. 7.**
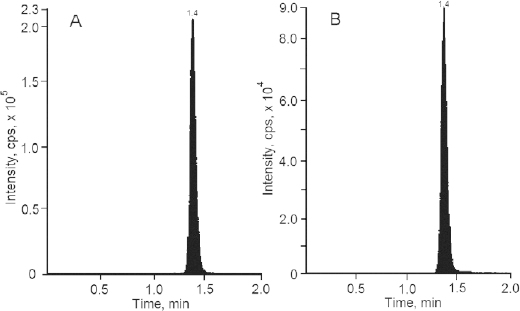
Representative chromatogram of highest calibration standard (A) zileuton and (B) zileuton D4

The LC-MS/MS method was selective for the intended analyte since the quantification was based on the mass-to-charge ratio of the parent as well as the product ion in MRM transition mode, which was selective and specific. The selectivity was also established for the blank plasma lots with the acceptance criteria of the analyte response, and the internal standard response observed in the blank plasma samples had to be less than 20% and 5% of the analyte and internal standard response of the LLOQ sample. No interference was observed for the blank plasma lots at the analyte and internal standard retention times. Figures 5, 6, and 7 represent the chromatogram of the blank plasma sample, lowest calibration standard, and highest calibration standards, respectively.

### Linearity

Considering the reported maximum plasma concentrations of around 4,400 ng/ml (Awni WM et al.), the linearity range of 50.5 ng/ml to 10,012.7 ng/ml was deemed suitable with the simple linear regression. Table 1 shows data from the calibration curves analysed for the evaluation of precision and accuracy during different days. The calibration curve includes eight calibration standards which are distributed throughout the calibration range. The correlation coefficient was considered for the evaluation of the goodness of fit. The average correlation coefficient was found to be 0.9978 with a goodness of fit. The details of the calibration curves are represented in Table 1.

**Tab. 1.** Precision and accuracy of calibration standards

Batch	STD 1	STD 2	STD 3	STD 4	STD 5	STD 6	STD 7	STD 8	Regression Coefficient (r value)
	Concentration (ng/ml)								
	50.5	100.9	360.5	801.0	2002.5	4005.1	8010.2	10012.7	
P&A-1	51.3	87.1	340.6	851.0	2100.6	4384.5	8478.6	10610.7	0.9992
P&A-2	51.2	88.6	343.2	775.6	2123.2	4427.6	8451.9	10527.1	0.9961
P&A-3	47.8	99.3	347.1	875.0	2164.1	4136.8	8276.6	10181.2	0.9982
Average	50.10	91.67	343.63	833.87	2129.3	4316.30	8402.37	10439.67	
Standard Deviation	1.992	6.653	3.272	51.868	32.186	156.938	109.732	227.708	0.9978
%RSD	4.0	7.3	1.0	6.2	1.5	3.6	1.3	2.2	

### Precision and Accuracy

Precision and accuracy were evaluated by analysing three precision and accuracy batches. Each precision and accuracy batch consisted of a calibration curve and six replicates of the LOQQC, LQC, MQC, and HQC. Precision and accuracy were evaluated both inter- and intra-batch. The intraday and interday precision and accuracy of the method for each zileuton concentration level (50.6, 148.8, 4,510.4, and 7,517.4 ng/ml) are presented in Table 2. The mean accuracy for each concentration level ranged from 102.1 to 107.0% and the mean of the relative standard deviation of precision for each concentration level ranged from 2.9 to 7.7%.

### Recovery

The recovery was evaluated by comparing the response of the extracted and unextracted samples. The extracted samples included six replicates of extracted LQC, MQC, and HQC samples. The unextracted samples included the aqueous solutions equivalent to the extracted samples. Internal standard recovery was evaluated in the same manner at the MQC level. The average recovery for zileuton in plasma ranged from 79.0 to 84.8% for the low, medium, and high quality control samples, respectively, with an average of 84.2%. The average recovery of the internal standard was 87.6%.

### Matrix Effect

The matrix effect was evaluated by processing two samples of blank plasma of six different lots till the extraction and the cartridges were extracted by equivalent aqueous solution at low (LQC) and high (HQC) quality control levels along with the internal standard. These samples were considered as post-extracted samples and were compared against the aqueous solutions. The matrix factor for the analyte and internal standard was calculated by comparing the peak response in the presence of the matrix ions to that of the peak response in the absence of the matrix ions. After calculation of the analyte and internal standard matrix factors separately, the internal standard normalized matrix factor was calculated by the ratio of the analyte/internal standard matrix factor. The mean internal standard normalized factor for zileuton at the low (LQC) and high (HQC) quality control samples ranged from 0.88 to 0.97.

### Stability Studies

Stability studies were performed to evaluate the stability of zileuton both in aqueous solution and in plasma after exposing it to various stress conditions. The stability studies performed include stock solution stability of zileuton and zileuton D4 in stock solution, stock dilution stability of zileuton in dilutions, benchtop stability in plasma, freeze-thaw stability in plasma, long-term storage stability in plasma, and autosampler stability of processed samples. All stability evaluations were performed as per international regulatory guidelines.

Zileuton and zileuton D4 stock solutions (2 mg/ml) remained stable when stored at refrigerator conditions for nine days, including the storage at room temperature for 8 h. Zileuton was stable in plasma samples when stored at room temperature for 18 h. Zileuton was found to be stable for three freeze and thaw cycles. Zileuton was stable and did not show any degradation when stored in the freezer for 74 days. Zileuton in the processed samples was stable for 38 h when stored in the autosampler at 4°C. The method characteristics are represented in Table 3.

**Tab. 2.** Between and within batch precision and accuracy of quality control samples (LLOQQC, LQC, MQC, and HQC) of three precision & accuracy batches

Nominal Concentration	LOQQC	LQC	MQC	HQC
	50.6	148.8	4510.4	7517.4
Precision and Accuracy Batch-1	47.4	156.8	4836.6	8077.0
	45.6	150.4	4922.9	8165.8
	54.5	160.5	4980.8	7835.9
	50.3	156.9	4649.2	8267.5
	52.3	157.7	4821.7	8066.2
	43.1	159.7	4509.2	8222.6
Average	48.87	157.00	4786.73	8105.83
Standard Deviation	4.281	3.568	176.552	154.034
%RSD	8.8	2.3	3.7	1.9
% Nominal	96.6	105.5	106.1	107.8

Precision and Accuracy Batch-2	52.6	165.2	5133.2	7983.3
	49.2	153.6	5181.8	7989.7
	51.5	160.3	4836.8	7514.8
	52.3	156.2	5056.6	8086.4
	52.5	163.6	5098.8	7874.2
	57.7	166.8	4786.1	8232.9
Average	25.63	160.95	5015.55	7946.88
Standard Deviation	2.788	5.221	164.131	243.478
%RSD	5.3	3.2	3.3	3.1
% Nominal	104.0	108.2	111.2	105.7

Precision and Accuracy Batch-3	53.1	158.4	4516.4	7494.6
	50.2	141.4	4219.2	8269.0
	58.0	145.7	4571.6	8152.9
	55.6	156.8	4500.0	8232.5
	55.8	156.9	4473.5	8079.8
	48.2	147.0	4405.3	8231.9
Average	53.48	151.03	4447.67	8076.78
Standard Deviation	3.717	7.204	124.484	293.256
%RSD	7.0	4.8	2.8	3.6
% Nominal	105.7	101.5	98.6	107.4

Global Precision and Accuracy				

Average	51.66	156.33	4749.98	8043.17
Standard Deviation	4.000	6.680	281.557	234.019
%RSD	7.7	4.3	5.9	2.9
% Nominal	102.1	105.1	105.3	107.0

**Tab. 3.** Method characteristics

Analyte	Zileuton
Internal Standard	Zileuton D4
Method Description	Liquid-liquid extraction
Regression Model	Linear regression with 1/Conc^2^ weighing
Analysis method	Peak area ratios
Limit of Quantification	50.5 ng/ml
Selectivity	No interference from the endogenous matrix components
Recovery of analyte	84.2%
Recovery of internal standard	87.6%
Linearity Range	50.5 to 10012.7 ng/ml
	LLOQQC: 50.6 ng/ml
	LQC: 148.8 ng/ml
Quality Controls concentrations	MQC: 4510.4 ng/ml
	HQC: 7517.4 ng/ml
QC interday accuracy range	102.1 to 107.0%
QC interday precision range	2.9 to 7.7
Benchtop stability	18 h at room temperature
Freeze-Thaw stability	3 cycles
Auto injector stability	38h
Long-term stability	74 days at -70°C
Stock solution stability	9 days
Stock dilution stability	26 h
Dilution integrity	1/2 and 1/4
Re-injection Reproducibility	One time

## Conclusion

Here we described the development of a simple, selective, precise, and accurate method for the quantification of zileuton in human plasma using a liquid chromatography-mass spectrometric method with the simple liquid-liquid extraction technique. For the quantification of zileuton, a smaller volume of plasma was used with the short run time of 2 minutes, thus making the method suitable for high throughput analysis during pharmacokinetic, bioequivalence, and drug interaction studies. The limit of quantification of the method was set to 50 ng/ml, considering the dosage of zileuton administered, and the linearity was established with simple linear regression. The method reported here uses a simple and effective extraction technique with good and reproducible recovery. The developed method was validated as per the current international regulatory guidelines on bioanalytical method validation.
